# PD-1 blockade induces remissions in relapsed classical Hodgkin lymphoma following allogeneic hematopoietic stem cell transplantation

**DOI:** 10.1186/s40425-017-0211-z

**Published:** 2017-02-21

**Authors:** James Godfrey, Michael R. Bishop, Sahr Syed, Elizabeth Hyjek, Justin Kline

**Affiliations:** 1 Department of Medicine, University of Chicago, Chicago, IL USA; 2 Department Pathology, University of Chicago, Chicago, IL USA

**Keywords:** Checkpoint blockade, PD-1, Hodgkin lymphoma, Allogeneic transplant

## Abstract

**Background:**

Allogeneic hematopoietic stem cell transplantation and checkpoint blockade therapy are immune-based therapies that have activity in selected refractory hematologic malignancies. Interest has developed in combining these treatments for high-risk hematologic diseases. However, there is concern that checkpoint blockade could augment graft-versus-host disease, and very few studies have evaluated the safety of checkpoint blockade in the post-allogeneic setting. Here, we report the outcomes of three patients with relapsed classical Hodgkin’s lymphoma following allogeneic transplant that were treated with the anti-PD-1 antibody, nivolumab.

**Case presentations:**

Three patients with Hodgkin’s lymphoma relapsed following allogeneic transplant received nivolumab therapy at our institution. All patients were free of graft-versus-host disease and were off of all systemic immunosuppressive medications at the time of nivolumab treatment. Nivolumab was well-tolerated in two of the patients. However, nivolumab had to be discontinued in one patient due to development of immune-related polyarthritis requiring treatment with systemic corticosteroids and methotrexate. Objective responses were observed in all three patients.

**Conclusions:**

Our case series demonstrates that anti-PD-1 therapy with nivolumab can be highly effective following allogeneic transplant for Hodgkin’s lymphoma, but serious immune-related adverse events can occur, requiring very close monitoring and interruption of therapy.

## Background

Allogeneic hematopoietic stem cell transplantation (alloHSCT) can be a curative treatment for high-risk and recurrent hematological malignancies [1]. A major therapeutic effect of alloHSCT lies within the graft-versus-tumor (GVT) response, where donor-derived lymphocytes recognize antigens expressed on the surface of malignant cells, and eliminate them from the host [2]. However, it has become clear that disease relapse following alloHSCT can be associated with immune evasion and loss of GVT effects [3]. Concrete examples include upregulation of programmed death-ligand 1 (PD-L1) on leukemia cells, and programmed death-1 (PD-1) on donor-derived T cells at the time of post-alloHSCT relapse, as well as deletion of human leukocyte antigen alleles in some leukemia patients relapsing after haploidentical alloHSCT [4, 5]. New strategies to restore GVT effects in these patients are needed because patients who relapse after alloHSCT have few treatment options and dismal outcomes [6].

Recently, the defined activity of checkpoint blockade therapy (CBT) with anti-PD-1 and anti-cytotoxic lymphocyte antigen-4 (CTLA-4) antibodies in a number of malignancies has generated interest in their use to treat disease relapse following alloHSCT. Preclinical studies of CBT have demonstrated augmentation of GVT effects [7–9], and two phase I studies of the anti-CTLA-4 antibody, ipilimumab, have been reported in patients with relapse after alloHSCT, where objective responses were observed [10, 11]. In the most recently published study, ipilimumab treatment was associated with a 31% objective response rate, including complete responses in patients with leukemia cutis, myeloid sarcoma, and myelodysplastic syndrome [11].

However, there is concern that enhancing the GVT effect through administration of CBT might also induce or exacerbate graft-versus-host disease (GVHD). Indeed, ipilimumab treatment was discontinued in 4 of 29 patients due to GVHD in the aforementioned study, and 6 patients had other immune-related adverse advents (IrAE) including one treatment-related death [11]. Further, PD-1 blockade has also been associated with induction of severe GVHD in murine models, and a report of fatal GVHD in a patient treated with the anti-PD-1 antibody, pembrolizumab, was recently published [12, 13]. These observations have somewhat tempered enthusiasm for the exploration of PD-1 blockade after alloHSCT to anecdotal reports and a retrospective case series which has been published in abstract form [14–16]. However, PD-1 blockade has been associated with a lower incidence of severe IrAE in non-transplant settings compared to CTLA-4 blockade, and is clearly more effective across a number of malignancies, including classical Hodgkin lymphoma (cHL) [17, 18]. We therefore sought to examine the safety and efficacy of PD-1 blockade following alloHSCT, and present data summarizing our experience with nivolumab for the treatment of relapsed cHL after alloHSCT.

## Case presentation

We treated three cHL patients who had relapsed after alloHSCT with off-label nivolumab at a dose of 3 mg/kg every 2 weeks. Patient and disease characteristics are summarized in Table 1. Briefly, all patients had multiply relapsed cHL despite treatment with conventional chemotherapy regimens and autologous hematopoietic stem cell transplantation. Patients received T cell-depleted grafts after reduced-intensity conditioning regimens. None developed acute GVHD, although two patients developed limited-stage chronic GVHD requiring short courses of steroids. Disease relapse occurred at an average of 1,008 days from alloHSCT (181, 389, and 2456 days), and was histologically confirmed in all cases. One patient received a donor lymphocyte infusion (DLI) at the time of relapse, but failed to achieve an objective response. After exhausting all conventional treatment options, patients were consented to treatment with nivolumab after discussing potential risks including life-threatening GVHD.

**Table 1:** Patient characteristics, adverse events, and response to nivolumab treatment

	Patient 1	Patient 2	Patient 3
Age	47	25	55
Sex	Male	Male	Male
Prior therapies (no.)	6	6	5
Stem cell source	Matched-related	Matched-related	Haploidentical and umbilical cord blood
Conditioning regimen	Reduced intensity	Reduced intensity	Reduced intensity
T cell depleted graft	Yes	Yes	Yes
Prior GVHD	No	Chronic GVHD of gut	Chronic oral GVHD
Days to relapse following AlloHSCT (no.)	181	2456	389
Localization and size of relapse	Diffuse bone and splenic involvement	Multifocal adenopathy in mediastinum, retroperitoneum and pelvis. Largest lymph node 2.3 × 1.5 cm in mediastinum	Multifocal adenopathy in neck, chest, abdomen and pelvis. Largest lymph node 4.2 × 1.8 cm in right axilla
Prior DLI	No	Yes	No
Immune-related adverse events	Grade 2 Keratoconjunctivitis	Grade 3 Inflammatory polyarthritis and grade 2 keratoconjunctivitis	Grade 1 Rash
Response to nivolumab	Partial response	Partial response	Partial response
Duration of response	6 Months+	10 Months+	14 Months+
Donor CD3^+^ chimerism before and after treatment	18 to 49%	Not available	Not available

**Fig. 1:**
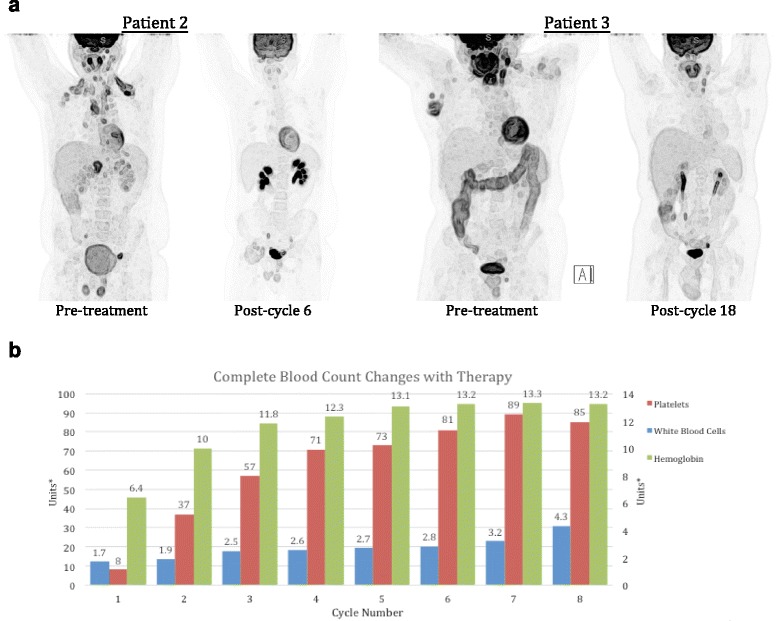
Radiographic and hematologic responses with therapy. **a** Response as assessed by sequential PET scan images. Images are shown for Patients 2 and 3. The response for Patient 1 was more difficult to illustrate on PET scan as his disease was primarily confined to the bone marrow. **b** Serial complete blood counts during treatment with nivolumab demonstrating significant tri-lineage improvement for Patient 1. *Platelet units (×10^3/uL) are provided on the left y-axis, while hemoglobin (g/dL) and white blood cell count units (×10^3/uL) are on the right y-axis

**Fig. 2:**
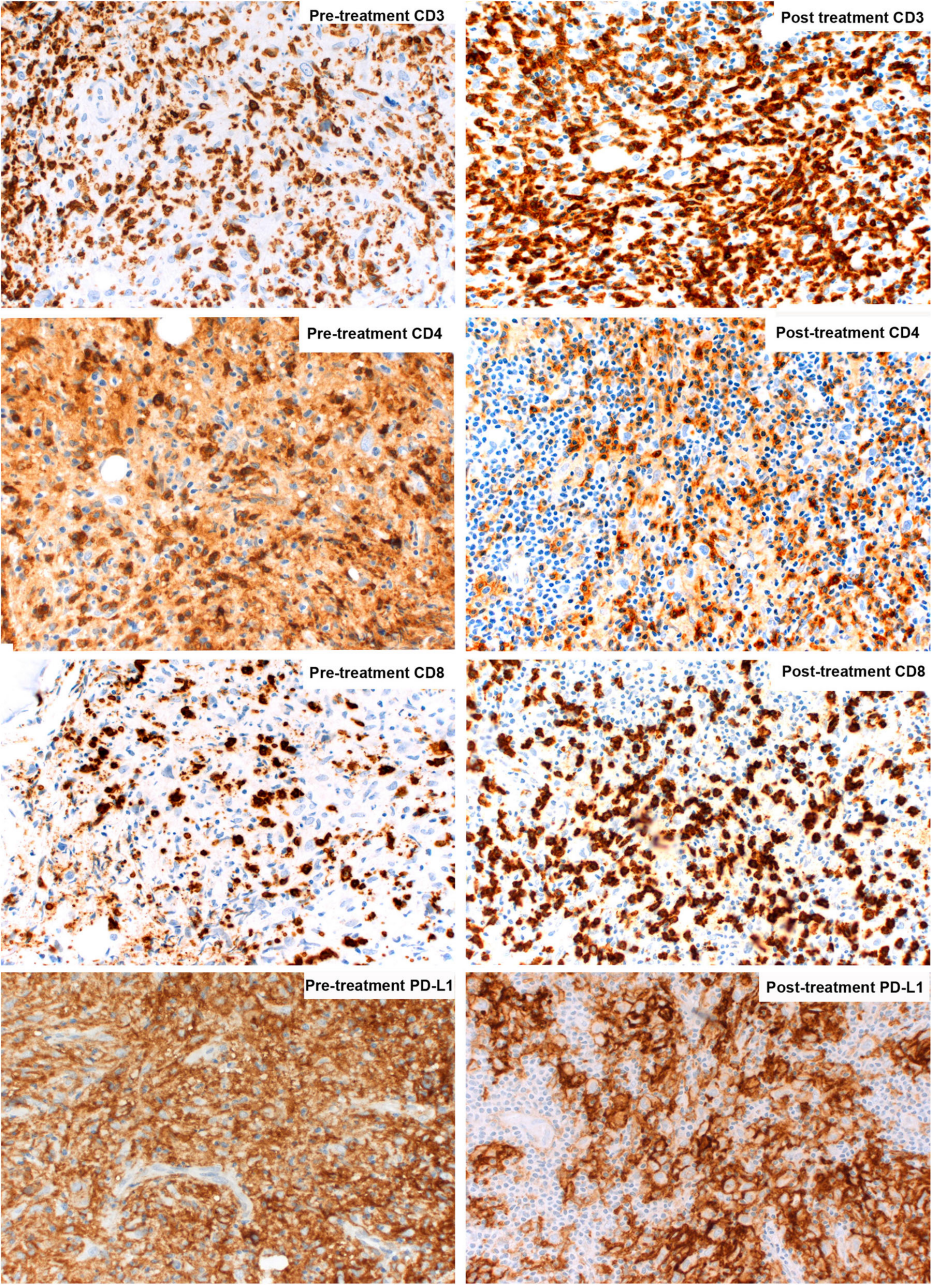
Immunohistochemistry changes with therapy. Immunohistochemistry results on bone marrow biopsies from patient 1 before and after 5 cycles of nivolumab. There is an increase in tumor-infiltrating CD8^+^ T cells and a decrease in CD4^+^ T cells with treatment. PD-L1 staining was positive both before and after treatment. Images were captured using a Jenoptik ProgRes® Speed XT core 5 series camera on an Olympus BX53 microscope at 50× magnification using Adobe Photoshop Software

All three patients had no clinical evidence of acute or chronic GVHD at the time of initiating nivolumab, and immunosuppressive medications had been discontinued at least a year prior to starting therapy. Observed nivolumab IrAE are listed in Table 1, and included grade 2 keratoconjunctivits in 2 patients, responsive to corticosteroid eye drops in both, grade 1 rash (possibly representing limited-stage chronic GVHD) in 1 patient, successfully treated with topical steroids, and one grade 3 episode of inflammatory polyarthritis, that required treatment discontinuation and administration of systemic corticosteroids and methotrexate to control. Mean duration of therapy is 8.3 months and is continuing in two patients. All patients had objective partial responses to treatment based on the results of interim PET scans (Fig. 1a). Responses are ongoing in all patients at the time of publication. In addition to radiographic responses, one patient with severe pancytopenia due to marrow involvement by cHL achieved a marked improvement in peripheral blood counts, as well as an objective improvement in bone marrow involvement by cHL (Fig. 1b). Immunohistochemistry studies on bone marrow biopsy samples before and after 5 cycles of nivolumab therapy in this patient demonstrated abundant PD-L1 expression in Hodgkin cells, with an increase in tumor-infiltrating CD8^+^ T cells and decreased CD4^+^ T cells with therapy (Fig. 2). Furthermore, bone marrow chimerism studies demonstrated an increase in the donor CD3^+^ compartment from 18 to 49% with treatment.

## Discussion and Conclusions

Our data conclusively demonstrate that anti-PD-1 therapy with nivolumab is effective for relapsed cHL following alloHSCT. All three patients have achieved objective and ongoing responses to treatment. These findings are consistent with previous observations demonstrating immune escape as a mechanism of relapse following alloHSCT, and that immune activating therapies such as DLI and CBT have the potential to restore GVT effects [3–5, 7–11, 19]. The finding of an objective response in a patient refractory to DLI, also demonstrates that PD-1 blockade reinvigorates GVT responses through distinct pathways that potentially more effectively activate GVT effects.

The toxicity of PD-1 blockade following alloHSCT will be an important measure to continue to assess in prospective studies. We did not observe any GVHD; however, several IrAEs occurred among the three patients reported here, none of which was life-threatening. It is expected, as has recently been reported [13], that the use of anti-PD-1 antibodies may result in severe immune-related toxicities in the post-allo-HSCT setting.

While our results are encouraging, it is not known whether other hematologic cancers will also be responsive to PD-1 blockade in the post-alloHSCT setting. That being said, complete responses to ipilimumab were observed after alloHSCT in patients with relapsed leukemia cutis and myelodysplastic syndrome [11]. This is an important observation since single-agent ipilimumab has only modest activity in hematologic malignancies outside of the alloHSCT setting [20], and may indicate that CBT and alloHSCT have therapeutically synergistic effects. In support of this, our finding of increased donor CD3^+^ chimerism at the site of disease involvement in one of our patients, suggests that anti-tumor effects are preferentially driven by donor-derived T cells following PD-1 blockade. Biologically, the enhanced activity of combining CBT with alloHSCT could be related to the observation that immune responses generated by PD-1 blockade are likely restricted to tumor neo-antigens in the non-transplanted host [21], whereas immune responses from donor-derived T cells are targeted against minor histocompatibility antigens that are more uniformly expressed [22]. Furthermore, anti-PD-1 antibodies may enhance the efficacy of alloHSCT by reinvigorating the GVT effect in hematological cancers that acquire PD-L1 expression as an adaptive response to immune pressure. Therefore the complementary immune effects of alloHSCT and PD-1 blockade hold promise in both the prevention and treatment of relapse following alloHSCT.

## Abbreviations

AlloHSCT: Allogeneic hematopoietic stem cell transplant
CBT: Checkpoint blockade therapy
cHL: Classical Hodgkin’s Lymphoma
CTLA-4: Cytotoxic T-lymphocyte associated protein-4
DLI: Donor lymphocyte infusion
GVHD: Graft-versus-host disease
GVT: Graft-versus-tumor
IrAE: Immune-related adverse event
PD-1: Programmed death receptor-1
PD-L1: Programmed death ligand-1
PET: Positron emission tomography
